# The Impact Imposed by Brand Elements of Enterprises on the Purchase Intention of Consumers—With Experience Value Taken as the Intermediary Variable

**DOI:** 10.3389/fpsyg.2022.873041

**Published:** 2022-06-09

**Authors:** Yi Zong, Menghui He

**Affiliations:** ^1^Postgraduate Office, Tianjin University of Commerce, Tianjin, China; ^2^School of Management, Tianjin University of Commerce, Tianjin, China

**Keywords:** brand elements, experience value, consumer purchase intention, intermediary variable, B2C

## Abstract

Against the backdrop of China's current dual-circulation paradigm, consumers have experienced the transition of their demands for products from price-driven needs to brand-driven needs. On the one hand, improving the value of consumer experience during brand building can enhance the exchanges and interactions between brands and consumers, facilitating the co-creation of value. On the other hand, the improvement of consumer experience can continuously strengthen the internal power of the construction of enterprise brand elements, and promote consumer-brand identity so as to constantly improve consumer purchase intention. In this study, we have taken consumers from the B2C market as the survey group while innovatively studying the impact imposed by brand elements on the purchase intention of consumers based on the experience value. In addition, we have established the theoretical framework of *brand elements*—*experience value*—*consumers' purchase intention* based on our analysis of the relationship between the aforementioned three factors. Through empirical research, we have elaborated on whether brand elements can improve consumers' purchase intention and whether the experience value can play an intermediary role. The research findings indicate that (1) the three dimensions of brand elements, namely, brand personality, brand value, and brand culture, can impose a significant positive impact on the purchase intention of consumers and experience value; (2) the specific parts of the experience value, namely, functional value, emotional value, social value, and service value, can impose a significant positive impact on the purchase intention of consumers; and (3) experience value can play an intermediary effect to some extent while brand elements influence the purchase intention of consumers.

## Introduction

As early as the 1960s, the American Marketing Association (AMA) proposed the definition of a brand, indicating that it is a name, proper noun, mark or symbol, or their combination, based on which most scholars extend from different fields and perspectives. More representative is the study by Aaker (1998) that pointed out that the brand is the consumers' overall perception and feeling of the products and services provided by the merchants, which is not only closely related to the characteristics of the product itself but also related to the enterprise itself, employee behavior, cultural concept, and consumers' personal preferences. The connotation of the brand has been deepened and expanded, leading to the concept of brand elements. By building the brand pyramid model, Harris and de Chernatony (2001) revealed that the components of the brand include five aspects: characteristics, interests, emotional return, values, and personality quality, which reflect the internal nature of the brand. Kotler et al. (2009), through the actual case research, pointed out that the overall brand components include brand characteristics, brand interests, brand personality, brand object, brand value, and brand culture. Wu and Zhou (2020) divided the brand image of an e-commerce platform into three dimensions: brand performance, brand personality, and corporate image of the e-commerce platform.

Kotler et al. (2009) pointed out that in the Internet era, the market has become increasingly open and networked, whereas the asymmetry in the information grasped by buyers and sellers has gradually diminished. Moreover, customers have experienced the transition of their needs for products from price-driven demands to quality and brand-driven ones, and they tend to opt for branded products, which has become the trend for the development of consumption, namely, consumption upgrading. In the process of brand factor construction, the trend of consumption upgrading leads to the improvement of customers' overall awareness of the brand. Abbott (1956) believed that what consumers really expect is not the product, but to get the experience in the process of consumer products. This experience is the customer's subject perception and value of everything experienced through the whole consumption process (Gan, 2019). It can be seen that the brand elements are closely related to the connotation of customer experience, and it can even be said that the brand is an experience of consumers.

In the Internet era, customer experience will spread to the outside at a geometric scale through modern marketing environments, such as the Internet, thus affecting others' consumption decisions and production or service performance (Tong et al., 2020). Experience marketing emerged at the moment. Compared with traditional marketing methods, experience marketing can improve brand value and customer loyalty by improving customers' experience during consumption (Yang, 2015). Experience marketing encourages consumers to use and feel the products and pays attention to interactive communication with consumers (Wang et al., 2021a). When customers get the consumer experience, they usually share this experience with others, thus creating product experience value, covering a range of internal and external experience value, such as functional value, emotional value, and social value (Tynan and McKechnie, 2009; Chen and Zhu, 2019; Li, 2019).

Therefore, this study believes that by shaping the perfect experience for consumers, brands can encourage consumers to take the initiative to pay a high premium and stay in the hearts of consumers in the way of sensory impressions, which can stimulate consumer purchase intention. In turn, improving the value of consumer experience can continuously strengthen the internal power of enterprise brand element construction, so it becomes more and more important to build a brand with the help of the consumer experience.

Through literature combing, a majority of the existing research has focused on the impact of a brand single element on consumer purchase intention (Chen et al., 2020a; Zhuang et al., 2021). As Sirgy and Su (2000) noted that the brand will have an impact on the decision-making of consumption behavior, including product preference and purchase intention. Zhu et al. (2015) proved that brand communication has a predictive effect on consumer purchase intention. Zhou et al. (2020) explored the influence of individual subjective status on the purchase intention of “servant” and “partner” anthropomorphic brands from the perspective of brand image. Baumert and de Obesso (2021) indicated that brand age has a statistically significant impact on consumer price setting. Çavuşoglu et al. (2021) examined the impact of brand image and brand awareness on perceived price and purchase intention. Therefore, it is not sufficient to study its influence on consumers' intention to buy from the overall perspective of brand elements.

Since the purchase process of consumers is a complex psychological activity, this study innovatively proposes that the brand should be regarded as a multi-level whole, and the composition of the brand and the influence mechanism of consumers from the overall perspective of consumer purchase intention. Especially, in the consumer experience scene, how can the brand elements ultimately affect the consumer purchase intention by affecting the value of the consumer experience? That is, will the experience value play a certain intermediary role between the brand elements and consumer purchase intention?

This study established the theoretical framework of “brand elements—experience value—consumers' purchase intention”. At the same time, different industries and brands may have different influences on consumers in brand elements, experience value perception, and purchase intention, so this study considers convenience, purchase, special products, and non-desire as control variables. Through empirical research, the study explores whether the improvement of brand elements can improve consumer purchase intention and whether the experience value can play an intermediary role in the two relationships. Finally, combined with the research conclusion, it is suggested that enterprise brand marketing should promote their purchase intention from the perspective of enhancing the value of the consumer experience.

## Theoretical Background Analysis and Research Hypotheses

### Brand Elements and Their Dimensions

The elements are the basic units of the constituent system, and the brand elements are the units contained by the brand. Generally speaking, a brand is composed of multiple elements. By combing the relevant literature, it is found that the brand elements mainly have three types, namely, dominant elements, recessive elements, and comprehensive elements (Huang, 2016; Shen et al., 2016b; Feng et al., 2017).

The dominant element of a brand is a tangible external performance of the brand, focusing on the sensory level of consumers (Gao et al., 2021; Zheng et al., 2021). The most representative figures are Park et al. (1991), who noted that the basic function of brand elements is to distinguish between ownership, including external elements, such as the name, image representation, advertising language, and packaging. Brand recessive elements are the performance of the internal attributes of the brand, such as cultural connotation, spiritual symbol, etc. Harris and de Chernatony (2001) revealed that the components of the brand include characteristics, interests, emotional returns, values, and personality quality, which are five aspects that reflect the internal essence of the brand. Kotler et al. (2009) pointed out the five aspects from the perspective of comprehensive elements, including brand characteristics, brand interests, brand personality, brand object, brand value, and brand culture. Li (2015) believed that the brand is generally composed of five basic elements, which include brand name, brand logo, brand concept, brand connotation, and brand objectives. Liu et al. (2016) distinguished brand elements more carefully. In the face of digital customized brands, she has established four elements, a total of 15 small elements. Zhao and Guo (2019) believed that the elements of the brand are divided into three categories: brand positioning, brand culture, and brand image. Büyükdag and Kitapci (2021) believed that the brand is influenced by elements, such as brand lifestyle consistency, brand identity, function, and social and emotional values.

Through the above literature, this study believes that the scholars proposed brand elements classification integration. Based on consumers' overall cognition of the brand, the dominant elements and recessive elements can be combined. On the basis of the overall brand division method of Kotler et al. (2009), we will guide enterprises to carry out more effective brand management from the perspective of comprehensive elements. A brand character includes brand characteristics, positioning, lifestyle, interests, image, characteristics, objectives, etc. Brand value includes brand interests, packaging, values, function, society, emotion, etc. Brand culture includes the brand name, image, positioning, identification, concept, connotation, etc. This study finally selects the three dimensions of brand character, brand value, and brand culture to constitute the brand elements, among which the brand character is the external embodiment of the brand personality; the brand value is the interests and functions transmitted by the brand to consumers, and the brand culture is the essential embodiment of the profound connotation of the brand.

### Experience Value Dimension Composition That Considers Brand Elements

With the arrival of the experience economy, the experience value has received widespread attention and research. Scholars' understanding of this concept is also deepening. Among them, the representative is the value co-creation theory based on customer needs. The customer experience value is the customer's cognition and evaluation of the degree to which their own needs are met (Butz and Goodstein, 1996; Cheng, 2007), which is the customer's diversified perception of the brand in the consumption process. This reflects the value demands of customers in the emotional, social, relationship, and other different aspects (Xu et al., 2015). The experience value is the relative psychological state and feedback of the product or service (Woodruff, 1997). Mathwick et al. (2001) believed that customers should get more happiness and emotional demands during the experience, which can not only affect their shopping goals but also improve the benefits of shopping. By creating self-perception priority, efficiency priority, and interactive priority experience scenarios, merchants have an impact on customers' psychological possession and control psychology and then affect customer value experience (Gao, 2021).

In order to quantify the above concept of experience value, scholars build the measurement dimensions of experience value from different perspectives.

Multidimensional classification method. Sheth et al. (1991) proposed that the experience value felt by customers includes functional value, social value, emotional value, cognitive value, and situational value. Hua et al. (2020) believed that the consumer level experience includes six dimensions: service improvement, social contact, psychological harvest, spiritual leisure, sharing, symbiosis, and altruistic service.

Introspection, association, and hierarchical division (Zhang and You, 2009). Based on the psychological theory, introspection divides consumer perception into perception challenges (Massimini and Carli, 1988). Association studies the relationship between customers and consumer scenarios from an internal or external, active, or passive perspective (Holbrook and Hirschman, 1982). The interaction between consumers and sellers affects the value of the experience (Pi and Liu, 2009). Mathwick et al. (2001) put forward four value dimensions: customer investment remuneration, service superiority, beauty, and interest. The hierarchical division makes experience value correspond to the five levels of physiological, safety, belonging, respect, and self-realization. Gentile et al. (2007) and Du and Fan (2007) divided the experience value into six dimensions: practicality, feeling, emotion, cognition, lifestyle, and association. Sweeney and Soutar (2001) and Shen et al. (2016a) divided the customer experience value into functional experience value, emotional experience value, and social experience value. Dai (2020) divided the experience value into instrumental value, spiritual value, emotional value, and social value. Wang et al. (2021b) believed that matching value, growth value, spiritual value, and interactive value are the newly emerging customer experience values in the online consumption situation.

The brand is the most important carrier of products. According to the above literature, the value of a product experience felt by customers is actually a degree of reaction to the brand. Braunsberger and Munch (1998) believed that consumer experience is obtained by contacting the brand through various activities. As the basic unit of the brand, each dimension may have an impact on customers' different experience value levels, which affect customers' purchase intention. Therefore, this study innovatively proposes that choosing the experience value division dimension combined with multi-dimensional and hierarchical levels can better verify that the experience value of the brand elements of different dimensions stimulates customer purchase intention.

This study, based on the existing literature, divides the experience value dimensions into four categories, namely, functional experience value, emotional experience value, social experience value, and service experience value. The basis of division mainly considers the consumers' demand factors for the product itself (the cognition of the product function and emotion) and the importance of the brand attributes (the diversified perception of the social attributes of the brand and the services provided).

### Impact Factors of Consumer Purchase Intention That Considers Brand Elements and Experience Value

As a behavioral trend, purchase intention is composed of consumers' attitudes, evaluation, and other factors (Jing and Shang, 2017), which reflect the psychological activities of consumers in making purchasing decisions in a specific environment. According to Blackwell and Miniard (2001), consumers make purchasing decisions on a particular brand based on finding information about an internal and external environment. Mao et al. (2020) focused on investigating consumer purchases through identity and brand-related structures. The results show that the flow experience, brand image, brand communication, brand personality, and brand identity all directly or indirectly explain the purchase intention. Flow experience plays a key intermediary role in the path from brand communication, brand character, and brand identity to purchase intention. Maharani et al. (2020) offered a proposal on factors that affect the willingness to buy products of self-owned brand products, in which in-store promotion, visual marketing, and in-store image directly affect customer value and purchase intention. Li and Zhang (2020) believed that consumer intention to repurchase is affected by their functional value, economic value, and perceived risk. Tong et al. (2020) believed that customer experience not only determines customers' own perception and satisfaction after consumption but also can spread outward at the speed of geometric series through modern marketing environments, such as the Internet, thus affecting others' consumption decisions and production or service performance. In addition, social responsibility (Ma, 2011; Chen, 2017), perceived value (Zhong, 2013), consumer trust (Escobar-Rodríguez and Bonsón-Fernández, 2017), and other factors will affect consumer purchase intention to some extent.

To sum up, consumer purchase intention is complex and will be affected by a variety of factors. Based on the previous literature, this article explores the influence on the purchase intention from two factors and multiple dimensions, which include brand elements and experience value. At the same time, it also focuses on age, education, gender, occupation, income, and commodity type of the variables.

### Research Hypothesis and Model Construction

To sum up, discussing the influence on consumers' purchase intention from the overall perspective of brand elements is not sufficient, especially in the context of considering various dimensions of experience value. This study aims at the blank points of previous research, grasps the experience value variables, and boldly puts forward relevant assumptions, with the purpose of verifying whether the improvement of brand elements can promote consumer purchase intention, and whether the experience value plays an intermediary role in the two relationships.

#### Brand Elements and Consumer Purchase Intention

As one of the most valuable intangible assets of an enterprise, the brand plays a role through the constituent elements. Sirgy and Su (2000) noted that brands will have an impact on consumer behavior decisions, including product preferences and willingness to buy. Zhu et al. (2015) proved that brand communication has a predictive effect on consumer purchase intention. Ma and Wang (2015) took cultural identity as the research perspective and examined the different effects of various forms of cultural identity on consumer purchase intention. An et al. (2020) pointed out that the brand image has a significant impact on consumer purchase intention, and consumers' brand identity plays an intermediary role. Du et al. (2020) also believed that consumers' perception of various dimensions of brand image positively affects their willingness to buy and share. Çavuşoglu et al. (2021) pointed out that there is a positive relationship between brand image and perceived price and purchase intention. To sum up, according to the existing studies, we can speculate that the brand elements will have an influence on consumer purchase intention, and it will have a positive influence.

Therefore, this study assumes H1: Brand elements can impose a positive impact on the purchase intention of consumers.

In addition, according to the dimensional classification of brand elements in this study, the existing literature is combed, speculating that the three elements including brand character, brand value, and brand culture will affect consumer purchase intention.

##### Brand Character

Wang and Zhang (2018) pointed out that the more consistent consumers' self-awareness and brand character are, the more is the consumers' willingness to buy. The results of Mao's (2020) modeling with the structural equations showed that flow experience, brand image, brand communication, brand character, and brand identity can all directly or indirectly explain the purchase intention. Flow experience plays a key intermediary role in the path from brand communication, brand character, and brand identity to purchase intention. Wu and Zhou (2020) believed that the brand character is one of the dimensions of the brand image of e-commerce platforms. The higher the consumer self-consistency, the stronger the impact of the brand character and the company image of the e-commerce platform on consumer purchase intention. Consumer self-consistency has no regulatory effect on the brand performance and purchase intention of e-commerce platforms.

Therefore, this study assumes H1a: Brand character can impose a positive impact on the purchase intention of consumers.

##### Brand Value

Wang et al. (2019) pointed out that both the functional value and emotional value of brands in rural tourism areas can significantly and positively affect tourists' willingness to revisit. Hua and Han (2020) believed that the functional value of the brand has a significant positive impact on customer purchase intention, and there is no difference between online and offline purchase channels in the impact of brand value on customer purchase intention. Petravičiute et al. (2021) showed that the greater the perceived value of luxury brands, the higher is the consumers' brand attachment, which results in the more willingness to buy. Meng (2021) pointed out that the necessity of improving the brand value of agricultural products in China is conducive to enhancing the market competitiveness of agricultural products and getting close to the target consumer groups so that consumers will be willing to pay for the products.

Therefore, this study assumes H1b: Brand value can impose a positive impact on the purchase intention of consumers.

##### Brand Culture

Tian et al. (2018) believed that the home textile brand culture is a cultural phenomenon of brand personalization, and the home textile brand culture has a profound impact on the consumption behavior of the target consumer groups. Wang (2020) believed that brand image culture is a symbol representing the enterprise, especially the establishment of brand culture image and value can make the brand stand out in the eyes of the market and consumers and affects consumption concept and even the social consumption culture. Chen et al. (2020b) showed that the information conveyed by brand culture can enable consumers to form a certain purchase tendency. Zhang (2020) believed that the cross-cultural integration strategy can help brands to better “enter” the host market and gain the recognition of local consumers, thus promoting the willingness of the host consumers to buy.

Therefore, this article assumes H1c: Brand culture can impose a positive impact on the purchase intention of consumers.

#### Brand Elements and Experience Value

The theoretical research of brand elements and experience started earlier. In terms of brand value, Braunsberger and Munch (1998) showed that the consumer experience of brand value is acquired through various activities, existing in the stages of brand recognition, purchase decision-making, and product use. In terms of brand culture, the results of Dirisu et al. (2018) showed that brand culture has a significant positive impact on the perceived experience value of the product. Quach et al. (2020) believed that experience value has a positive impact on both brand attitude and purchase engagement. The more the customers pay attention to privacy, the stronger the impact of experience value on the brand attitude. In terms of brand character, Jin et al. (2012) pointed out that luxury brands bring consumers a good functional experience and high social value, as well as emotional and service experience. When studying service industry brands, Yang (2014) combined the brand character with experience to obtain the application of the law of brand clothing in experience design. Zhang (2019) confirmed that the brand will improve customer purchase intention and explained that the brand-related stimuli make consumers obtain psychological and behavioral experience perception, including brand recognition, brand image, communication methods, and marketing environment. Xu et al. (2021) believed that only by fully guiding consumers' brand interaction and mobilizing and enhancing consumers' brand experience, can the brand value be effectively realized.

To sum up, it can be seen that the conscious strengthening and promotion of various dimensions of brand elements (brand character, brand value, and brand culture) can make consumers perceive the acquisition of multi-level experience value, such as high quality (functional value), social status (social value), emotional and sensory experience (emotional value), service perception (service value), etc. Thus, this study puts forward Hypothesis H2: Brand elements can impose a positive impact on the experience value. On the basis of proposing H2, the correspondence is subdivided from the respective constituent dimensions of brand elements and experience value to further propose the following assumptions:

H2a: Brand character, brand value, and brand culture can, respectively, impose positive impacts on the functional value.
H2b: Brand character, brand value, and brand culture can, respectively, impose positive impacts on emotional value.
H2c: Brand character, brand value, and brand culture can, respectively, impose positive impacts on social value.
H2d: Brand character, brand value, and brand culture can, respectively, impose positive impacts on the service value.

#### Experience Value and Consumer Purchase Intention

Mathwick et al. (2001) verified that the experience value will have a positive impact on brand preference and repeat intention during the operation of the retail store. When studying the value of the online shopping experience, Sun (2018) pointed out that satisfaction and social experience perception can improve consumers' happiness. Zhao (2019) studied the relationship between retail brand image, perceived experience value, and consumer care behavior, and found that both quality image and commodity display image can positively affect consumer perceived value. In addition to cognitive value, functional value, emotional value, and economic value can significantly promote consumer care behavior. Tang et al. (2021) pointed out that both brand marketing stimulation and external stimulation have a significant positive impact on experience marketing motivation. Then, experience marketing motivation has a significant positive impact on purchase intention.

To sum up, it can be inferred that the perception of experience value (including functional, emotional, social, and service value) can stimulate consumers' purchase behavior to a certain extent and positively affect their purchase intention. So, this hypothesis is proposed:

H3: Experience value can impose a positive impact on the purchase intention of consumers.
H3a: Functional value can impose a positive impact on the purchase intention of consumers.
H3b: Emotional value can impose a positive impact on the purchase intention of consumers.
H3c: Social value can impose a positive impact on the purchase intention of consumers.
H3d: Service value can impose a positive impact on the purchase intention of consumers.

#### Intermediary Role Played by the Experience Value

The experience value is an important factor in the experience economy and the main content of scholars' research. Shen et al. (2016a), Li (2017), and Liu (2019) studied the impact of customer interaction on value co-creation based on the experience value as the intermediary variable in the online community and industrial tourism. Kuang (2019) proved that the service quality and the tourist experience value will affect the behavior intention of the tourists in the scenic spots, and the experience value can play a positive intermediary role between the two variables. Gao (2021) believed that only considering sales tasks and self-feelings, regardless of consumer feelings, will reduce customers' evaluation of the products, and a good service relationship can increase customers' acceptance of the products. According to the relationship between brand elements and consumer experience value, it can be seen that consumers' cognition of brand elements is obtained and deepened through brand experience in various forms of activities. By experiencing this behavioral stimulus, consumers will feel the advantages of the brand in terms of function, service, and emotional and social value, which has a positive impact on consumer preference and willingness to repeat purchases. Mao et al. (2020) noted that the flow experience plays a key intermediary role in the path from brand communication, brand character, and brand identity to the purchase intention. Therefore, this study speculated that experience value may influence brand elements and consumer purchase intention and proposed the hypothesis of this article.

H4a: Functional value can play an intermediary role in the relationship between brand elements and the purchase intention of consumers.
H4b: Emotional value can play an intermediary role in the relationship between brand elements and the purchase intention of consumers.
H4c: Social value can play an intermediary role in the relationship between brand elements and the purchase intention of consumers.
H4d: Service value can play an intermediary role in the relationship between brand elements and the purchase intention of consumers.

### Research Model Construction

**Figure 1:**
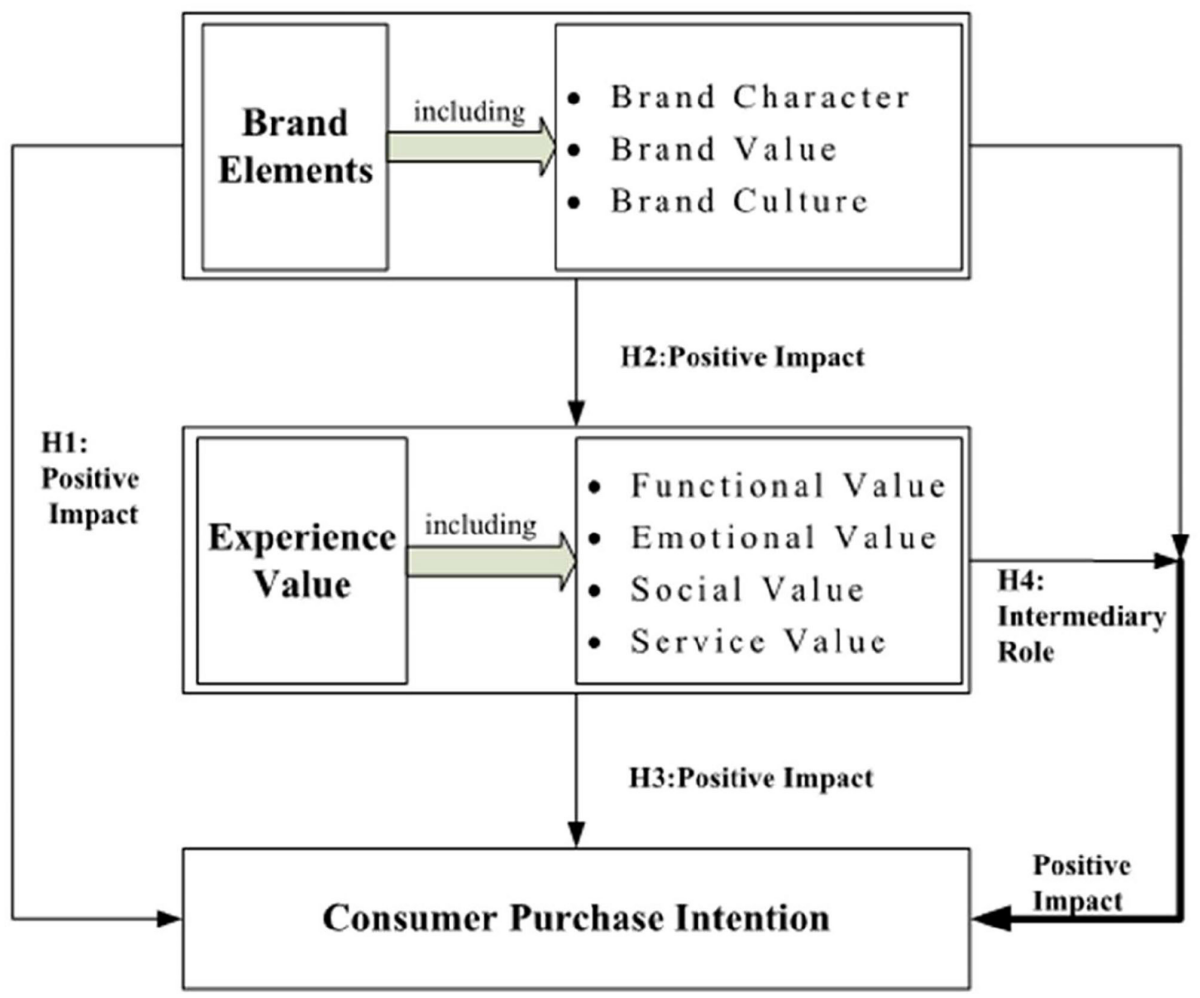
Model of theoretical research.

Based on the hypothesis, the research model of this article is constructed as follows (Figure 1), including the following four parts:

(1) Research on the relationship between brand elements and consumer purchase intention.

(2) Research on the relationship between brand elements and experience value, in which the brand is divided into three dimensions, namely brand culture, brand value, and brand character, and the experience value is divided into four dimensions: functional value, emotional value, social value, and service value.

(3) Research on the relationship between experience value and consumer purchase intention.

(4) The mediation role of experience value.

## Research Design

### Selection of Research Methods

According to the model construction illustrated in Figure 1, based on the research on the relationship between brand elements, experience value, and purchase intention, this article integrates the existing theories and relevant research, infers the relationship between variables, puts forward research hypotheses, and selects the research method of combining questionnaire survey method and empirical research method for hypothesis verification.

With the B2C consumer market as the main investigation body, the corresponding questionnaire was designed based on the research results of brand elements, experience value, and consumer purchase intention. The content of the questionnaire survey is to understand the consumers “views of the brand in the purchase behavior and whether the brand affects the purchase behavior, the influence of the brand elements on the consumers” purchase willingness, and the intermediary role of the experience value.

We analyzed the data obtained through the questionnaire, set the regression model according to the research hypothesis and the alternative interpretation that needs to be excluded, then verified or tested the research hypothesis through robust statistical analysis, systematically explained the analysis results, and discussed the relationship between verifying research hypothesis and verifying theory both theoretically and logically (Liang, 2021). In this study, the correlation analysis was selected to describe the relationship between the variables to lay the foundation for the next causality test. On the basis of the correlation test, in order to further study the causal relationship and connection strength between variables and verify the model hypothesis, regression analysis was carried out on each variable in this article to test whether the model is effective. Finally, the mediation effect validation was performed, through which way the independent variable affects the dependent variable, which belongs to the analysis of causal transmission. In the output results, the first display is the results of the first regression equation, focusing on whether the regression coefficient is significant. The second result reflects the regression coefficient of the intermediary equation. Therefore, we can partially judge whether the intermediary effect exists. Finally, the relationship between brand elements, experience value, and purchase intention are confirmed, and the operation rules are summarized to provide a basis for brand marketing.

### Scale Selection and Questionnaire Design

Starting with the eight variables of brand character, brand value, brand culture, functional value, emotional value, social value, service value, and consumer purchase intention, based on the mature scale at home and abroad, combined with the appropriate modifications needed to be made in this article, the measurement scale is established to form a questionnaire.

#### Brand Elements Measurement Scale

Based on the studies by Dong (2019), Keller (2003), Altaf et al. (2017), and Huang (2016), the brand character dimension is set as A1i (*i* = 1~3), brand value dimension as A2i (*i* = 1~4), and brand culture dimension as A3i (*i* = 1~5) (shown in Supplementary Table 1).

#### Experience Value Measurement Scale

Based on the research studies by Sweeney and Soutar (2001), Cheng (2010), Holbrook (2006), Zou et al. (2007), and other scholars, the functional value dimension is set as B1i (*i* = 1~4), emotional value dimension is set as B2i (*i* = 1~4), social value dimension is set as B3i (*i* = 1~4), and service value dimension is set as B4i (*i* = 1~4) (shown in Supplementary Table 2).

#### Consumer Purchase Intention Measurement Scale

Based on the study by Anckar and D'Incau (2002), the dimension was set to three questions: C1i (*i* = 1~3) (shown in Supplementary Table 3).

#### Questionnaire Design

The questionnaire was composed of four parts. The first section provides statistics on basic information. The second, third, and fourth parts designed the questionnaire according to the question items of brand elements, experience value, and purchase intention, respectively, and then the 5-point Likert scale scoring method was adopted in the research process (1–5 indicates very non-conforming, non-conforming, general, conforming, and fully conforming). The reason why it is divided into five options mainly considers that the different scores subjectively given by the questionnaire designer and the assignments considered by the respondents, so that the measurement results cannot fully reflect the true attitude of the respondents in some cases, and thus affecting the reliability of the statistical analysis results. Using the five-point method can make the option more fully close to the subjective feelings of consumers than the three-point method, making it easy to choose a closer option to the actual option. In particular, the customer purchase intention as a qualitative variable adopts quantitative processing of the five-point method, which can objectively and reasonably reduce the difference of data, so as to improve the effectiveness and accuracy of the data. A total of 375 questionnaires were issued, and 330 valid questionnaires were obtained excluding invalid questionnaires. The recovery rate reached 88%.

### Sample Structure and Design

This study takes the B2C consumer market as the research sample, which mainly includes gender, age, education, occupation, monthly income (Yuan), and purchase of brand goods. The selected research samples cover different genders, ages, education, occupation, monthly incomes (Yuan), and other aspects, representing the cognition and attitudes of different groups toward the brand. The sample age basically covers the age level with a strong consumption level, mainly between 18 and 35 years old. Most of the sample degrees are bachelor's and master's degrees, with a high knowledge level, which can ensure the accuracy and reliability of the questionnaire filling. The sample occupation and monthly income were relatively even with more or <3,500 yuan, probably due to the larger student population in the sample. From the perspective of the types of goods, consumers pay more attention to the brand when buying shopping products and special products. In conclusion, the sample structure distribution is relatively balanced, meeting the requirements for the sample data in this study.

## Data Analysis and Hypothesis Test

### Analysis of Scale Reliability and Validity

#### Reliability

The measured sample coefficients of each dimension are specified in Supplementary Table 4. Cronbach's Alpha coefficient of each dimension amounted to above 0.80, indicating that each scale featured high reliability and strong stability.

#### Validity

##### Validity Test of Brand Element Scale

From Supplementary Table 5, the KMO of brand elements amounted to 0.845, the approximate Chi-square of Bartlett's test fell within an appropriate range, whereas the *P*-value amounted to 0.000, indicating that we may further conduct the exploratory factor analysis on the brand element scale.

We conducted the factor load analysis of brand elements. From Supplementary Table 6, the factor load coefficient of each dimension all exceeded 0.8, indicating that the measurement questions for each dimension of brand elements can meet the requirements with strong interpretation capability and optimal validity.

##### Validity Test of the Experience Value Scale

From Supplementary Table 7, the KMO of experience value amounted to 0.835, the approximate Chi-square of Bartlett's test fell within an appropriate range, whereas the *P*-value amounted to 0.000, indicating that we may further conduct the exploratory factor analysis on the experience value scale.

We conducted the factor load analysis of experience value. From Supplementary Table 8, we found that the factor load coefficient of each dimension of experience value all exceeded 0.8, indicating that the measurement questions of each dimension of experience value can meet the requirements with strong interpretation capability and optimal validity.

##### Validity Test of Consumers' Purchase Intention Scale

From Supplementary Table 9, the KMO of consumers' purchase intention amounted to 0.704, the approximate Chi-square of Bartlett's test fell within an appropriate range, whereas the *P*-value amounted to 0.000, indicating that we may further conduct the exploratory factor analysis on the consumers' purchase intention scale.

We conducted the factor load analysis of the purchase intention of consumers. From Supplementary Table 10, we found that the load factor coefficient of each dimension of consumers' purchase intention all exceeded 0.8, indicating that the measurement questions of each dimension of consumers' purchase intention can meet the requirements with strong interpretation capability and optimal validity.

### Analysis of Correlation Coefficient

Correlation analysis is mainly used to describe the correlation among variables. The Pearson's correlation coefficient is used to analyze the degree of correlation among brand elements, experience value, and consumer purchase intention (Table 1).

**Table 1:** Analysis of correlation coefficient.

		**Brand** **character**	**Brand** **value**	**Brand** **culture**	**Functional** **value**	**Emotional** **value**	**Social** **value**	**Service** **value**	**Consumers'** **purchase** **intention**
Brand character	Pearson correlation	1							
	Significance (double tail)								
Brand value	Pearson correlation	0.135^*^	1						
	Significance (double tail)	0.014							
Brand culture	Pearson correlation	0.159^**^	0.210^**^	1					
	Significance (double tail)	0.004	0.000						
Functional value	Pearson correlation	0.407^**^	0.384^**^	0.496^**^	1				
	Significance (double tail)	0.000	0.000	0.000					
Emotional value	Pearson correlation	0.335^**^	0.344^**^	0.351^**^	0.258^**^	1			
	Significance (double tail)	0.000	0.000	0.000	0.000				
Social value	Pearson correlation	0.437^**^	0.400^**^	0.351^**^	0.187^**^	0.227^**^	1		
	Significance (double tail)	0.000	0.000	0.000	0.001	0.000			
Service value	Pearson correlation	0.397^**^	0.478^**^	0.267^**^	0.211^**^	0.169^**^	0.271^**^	1	
	Significance (double tail)	0.000	0.000	0.000	0.000	0.002	0.000		
Consumers'	Pearson correlation	0.338^**^	0.385^**^	0.347^**^	0.414^**^	0.362^**^	0.428^**^	0.393^**^	1
purchase intention	Significance (double tail)	0.000	0.000	0.000	0.000	0.000	0.000	0.000	

* *The correlation is significant at the significance level of 0.05 (double tail)*.

** *The correlation is significant at the significance level of 0.01 (double tail)*.

From Table 1, we found that there was a certain correlation between the three dimensions of brand elements and the purchase intention of consumers, between the three dimensions of brand elements and the experience value, and between the experience value and the purchase intention of consumers. In addition, there was a significant correlation between the variables at the significance level of 0.01. Therefore, we came to the preliminary conclusion that the research hypotheses can impose a positive impact.

### Regression Analysis

On the basis of the correlation test of each variable, the significance test was conducted, and the regression analysis was selected to verify the model hypothesis. The available data were characterized by normality, independence, linear correlation, and homoscedasticity, which satisfied the Gaus Markov assumptions, so the regression analysis could be applied. The independent variable was set as each dimension of brand elements, the intermediary variable was set as each dimension of experience value, and the dependent variable was set as the consumer purchase intention to test the regression relationship of the above variables.

#### Regression Analysis of Brand Elements to Consumers' Purchase Intention

##### Regression Analysis of Brand Elements to Consumers' Purchase Intention

The regression analysis was conducted with the overall brand elements as the independent variable in the regression equation and the consumer purchase intention in the regression equation, and the results are shown in Table 2.

**Table 2:** Regression analysis of brand elements to consumers' purchase intention.

	**Model 1**	**Unstandardized coefficient**		**Standardized coefficient**	***t***	**Significance**
		**B**	**Standard error**	**Beta**		
Independent variable	(Constant)	1.284	0.410		3.128	0.002
	Brand element	0.575	0.063	0.462	9.107	0.000
Control variable	Student	0.100	0.118	0.059	0.847	0.397
	Civil servant and public institution personnel	0.082	0.145	0.033	0.566	0.571
	Enterprise personnel	0.343	0.126	0.173	2.711	0.007
	Businessman	0.578	0.176	0.181	3.273	0.001
	Others	0				
	Convenience goods	0.040	0.207	0.016	0.195	0.845
	Shopping goods	−0.036	0.248	−0.022	−0.146	0.884
	Specialty goods	−0.058	0.157	−0.031	−0.370	0.712
	Unsought goods	0				
	Gender	0.052	0.080	0.031	0.647	0.518
	Monthly income	0.012	0.080	0.017	0.148	0.882
*R*		0.571				
*R* ^2^		0.326				
Adjusted *R*^2^		0.305				
F		15.454				
Sig		0.000				

*The dependent variable is the consumers' purchase intention*.

From Table 2, we inferred that the standardized coefficient of brand elements amounted to 0.462, which was a positive value with a significance level of 0.000. The results showed that brand elements can play a significant role in enhancing the purchase intention of consumers. Therefore, the H1 has been verified.

We have obtained the linear regression equation for Model 1 as follows:

Consumers' Purchase Intention = 0.462 ^*^ Brand Elements + 0.173 ^*^ Enterprise Personnel + 0.181 ^*^ Businessman + ε (Formula 1).

##### Regression Analysis of the Dimensions of Brand Elements to Consumers' Purchase Intention

The three dimensions of brand character, brand value, and brand culture of brand elements are taken as the independent variables, and consumer purchase intention is returned as the dependent variable. The results are shown in Table 3.

**Table 3:** Regression analysis of the dimensions of brand elements to consumers' purchase intention.

	**Model 2**	**Unstandardized coefficient**		**Standardized coefficient**	***t***	**Significance**
		**B**	**Standard error**	**Beta**		
Independent variable	(Constant)	1.275	0.412		3.098	0.002
	Brand character	0.180	0.040	0.219	4.512	0.000
	Brand value	0.219	0.041	0.265	5.379	0.000
	Brand culture	0.177	0.041	0.209	4.280	0.000
Control variable	Student	0.108	0.119	0.064	0.907	0.365
	Civil servant and public institution personnel	0.095	0.147	0.038	0.647	0.518
	Enterprise personnel	0.349	0.127	0.176	2.748	0.006
	Businessman	0.586	0.177	0.184	3.303	0.001
	Others	0				
	Convenience goods	0.042	0.208	0.016	0.202	0.840
	Shopping goods	−0.034	0.249	−0.020	−0.136	0.892
	Specialty goods	−0.061	0.157	−0.033	−0.387	0.699
	Unsought goods	0				
	Gender	0.052	0.081	0.031	0.652	0.515
	Monthly income	0.012	0.080	0.017	0.145	0.885
*R*		0.572				
*R* ^2^		0.328				
Adjusted *R*^2^		0.302				
F		12.877				
Sig		0.000				

*The dependent variable is the consumers' purchase intention*.

From Table 3, we concluded that the standardized coefficient of the three dimensions of brand elements amounted to 0.219, 0.265, and 0.209, respectively, which were all positive values with a significance level of 0.000. The results showed that the three dimensions of brand elements can play a significant role in enhancing consumers' purchase intention. Therefore, H1a, H1b, and H1c have been verified.

We obtained the linear regression equation for Model 2 as follows:

Consumers' purchase intention = 0.219 ^*^ Brand Character + 0.265 ^*^ Brand Value + 0.209 ^*^ Brand Culture + 0.176 ^*^ Enterprise Personnel + 0.184 ^*^ Businessman + ε (Formula 2).

#### Regression Analysis of Brand Elements to Experience Value

The three dimensions of brand elements, brand character, brand value, and brand culture, were taken as the independent variables, and the experience value was returned as the dependent variables. The results are shown in Table 4.

**Table 4:** Regression analysis of brand elements to experience value.

	**Model 3**	**Unstandardized coefficient**		**Standardized coefficient**	***t***	**Significance**
		**B**	**Standard error**	**Beta**		
Independent variable	(Constant)	0.208	0.177		1.175	0.241
	Brand elements	0.899	0.027	0.887	32.975	0.000
Control variable	Student	0.071	0.051	0.052	1.391	0.165
	Civil servant and public institution personnel	0.023	0.063	0.011	0.370	0.712
	Enterprise personnel	0.123	0.055	0.076	2.255	0.025
	Businessman	0.089	0.076	0.034	1.161	0.247
	Others	0				
	Convenience goods	−0.034	0.089	−0.016	−0.377	0.706
	Shopping goods	−0.088	0.107	−0.064	−0.816	0.415
	Specialty goods	−0.038	0.068	−0.025	−0.566	0.572
	Unsought goods	0				
	Gender	−0.007	0.035	−0.005	−0.215	0.830
	Monthly income	−0.025	0.035	−0.044	−0.711	0.478
*R*		0.901				
*R* ^2^		0.811				
Adjusted *R*^2^		0.805				
F		136.907				
Sig		0.000				

*The dependent variable is the experience value*.

##### Regression Analysis of Brand Elements to Experience Value

From Table 4, we concluded that the standardized coefficient of brand elements amounted to 0.887, which was a positive value with a significance level of 0.000. The results showed that the three dimensions of brand elements can play a significant role in enhancing the experience value. Therefore, H2 has been verified.

We obtained the linear regression equation for Model 3 as follows:

Experience Value = 0.887 ^*^ Brand Elements + 0.076 ^*^ Enterprise Personnel + ε (Formula 3).

##### Regression Analysis of Each Dimension of Brand Elements to Functional Value

From Table 5, we concluded that the standardized coefficient of the three dimensions of brand elements amounted to 0.300, 0.258, and 0.388, respectively, which were positive values with a significance level of 0.000. The results showed that the three dimensions of brand elements can play a significant role in enhancing the functional value. Therefore, H2a has been verified.

**Table 5:** Regression analysis of each dimension of brand elements to functional value.

	**Model 4**	**Unstandardized coefficient**		**Standardized coefficient**	***t***	**Significance**
		**B**	**Standard error**	**Beta**		
Independent variable	(Constant)	0.168	0.451		0.372	0.71
	Brand character	0.293	0.044	0.300	6.715	0.000
	Brand value	0.254	0.045	0.258	5.704	0.000
	Brand culture	0.389	0.045	0.388	8.612	0.000
Control variable	Student	0.116	0.131	0.058	0.888	0.375
	Civil servant and public institution personnel	0.020	0.161	0.007	0.126	0.900
	Enterprise personnel	0.245	0.139	0.104	1.764	0.079
	Businessman	0.234	0.194	0.061	1.202	0.230
	Others	0				
	Convenience goods	−0.067	0.227	−0.022	−0.297	0.767
	Shopping goods	−0.133	0.273	−0.066	−0.486	0.627
	Specialty goods	−0.092	0.172	−0.041	−0.532	0.595
	Unsought goods	0				
	Gender	0.057	0.088	0.028	0.645	0.519
	Monthly income	−0.069	0.088	−0.085	−0.788	0.431
*R*		0.657				
*R* ^2^		0.431				
Adjusted *R*^2^		0.410				
F		20.019				
Sig		0.000				

*The dependent variable is the functional value*.

We obtained the linear regression equation for Model 4 as follows:

Functional Value = 0.300 ^*^ Brand Character + 0.258 ^*^ Brand Value + 0.388 ^*^ Brand Culture + ε (Formula 4).

##### Regression Analysis of Each Dimension of Brand Elements to Emotional Value

From Table 6, we concluded that the standardized coefficient of the three dimensions of brand elements amounted to 0.245, 0.251, and 0.230, respectively, which were all positive values with a significance level of 0.000. The results showed that the three dimensions of brand elements can play a significant role in enhancing emotional value. Therefore, H2b has been verified.

**Table 6:** Regression analysis of each dimension of brand elements to the emotional value.

	**Model 5**	**Unstandardized coefficient**		**Standardized coefficient**	***t***	**Significance**
		**B**	**Standard error**	**Beta**		
Independent variable	(Constant)	0.054	0.549		0.099	0.922
	Brand character	0.260	0.053	0.245	4.891	0.000
	Brand value	0.269	0.054	0.251	4.960	0.000
	Brand culture	0.251	0.055	0.230	4.558	0.000
Control variable	Student	0.219	0.159	0.100	1.377	0.169
	Civil servant and public institution personnel	0.491	0.196	0.153	2.509	0.013
	Enterprise personnel	0.325	0.169	0.127	1.919	0.056
	Businessman	0.525	0.236	0.127	2.219	0.027
	Others	0				
	Convenience goods	−0.230	0.277	−0.069	−0.831	0.407
	Shopping goods	0.033	0.332	0.015	0.100	0.920
	Specialty goods	−0.007	0.210	−0.003	−0.035	0.972
	Unsought goods	0				
	Gender	0.065	0.107	0.030	0.609	0.543
	Monthly income	0.004	0.107	0.004	0.036	0.972
*R*		0.536				
*R* ^2^		0.287				
Adjusted *R*^2^		0.260				
F		10.653				
Sig		0.000				

*The dependent variable is the emotional value*.

We obtained the linear regression equation for Model 5 as follows:

Emotional Value = 0.245 ^*^ Brand Character + 0.251 ^*^ Brand Value + 0.230 ^*^ Brand Culture + 0.153 ^*^ Civil servant and public institution personnel + 0.127 ^*^ Businessman+ ε (Formula 5).

##### Regression Analysis of Each Dimension of Brand Elements to Social Value

From Table 7, we concluded that the standardized coefficient of the three dimensions of brand elements amounted to 0.348, 0.283, and 0.221, respectively, which were all positive values with a significance level of 0.000. The results showed that the three dimensions of brand elements can play a significant role in enhancing social value. Therefore, H2c has been verified.

**Table 7:** Regression analysis of each dimension of brand elements to social value.

	**Model 6**	**Unstandardized coefficient**		**Standardized coefficient**	***t***	**Significance**
		**B**	**Standard error**	**Beta**		
Independent variable	(Constant)	−0.138	0.543		−0.254	0.800
	Brand character	0.390	0.053	0.348	7.416	0.000
	Brand value	0.318	0.054	0.283	5.935	0.000
	Brand culture	0.254	0.054	0.221	4.669	0.000
Control variable	Student	−0.061	0.158	−0.027	−0.388	0.698
	Civil servant and public institution personnel	−0.073	0.194	−0.022	−0.378	0.706
	Enterprise personnel	−0.065	0.167	−0.024	−0.387	0.699
	Businessman	−0.037	0.234	−0.008	−0.156	0.876
	Others	0				
	Convenience goods	0.214	0.274	0.061	0.780	0.436
	Shopping goods	−0.017	0.328	−0.008	−0.053	0.958
	Specialty goods	0.072	0.208	0.028	0.346	0.729
	Unsought goods	0				
	Gender	−0.064	0.106	−0.028	−0.607	0.545
	Monthly income	0.074	0.106	0.079	0.693	0.489
*R*		0.609				
*R* ^2^		0.371				
Adjusted *R*^2^		0.348				
F		15.61				
Sig		0.000				

*The dependent variable is the social value*.

We obtained the linear regression equation for Model 6 as follows:

Social Value = 0.348 ^*^ Brand Character + 0.283 ^*^ Brand Value + 0.221 ^*^ Brand Culture + ε (Formula 6).

##### Regression Analysis of Each Dimension of Brand Elements to Service Value

From Table 8, we concluded that the standardized coefficient of the three dimensions of brand elements amounted to 0.344, 0.429, and 0.155, respectively, which were all positive values with a significance level of 0.000. The results showed that the three dimensions of brand elements can play a significant role in enhancing the service value. Therefore, H2d has been verified.

**Table 8:** Regression analysis of each dimension of brand elements to service value.

	**Model 7**	**Unstandardized coefficient**		**Standardized coefficient**	***t***	**Significance**
		**B**	**Standard error**	**Beta**		
Independent variable	(Constant)	0.694	0.477		1.455	0.147
	Brand character	0.341	0.046	0.344	7.390	0.000
	Brand value	0.428	0.047	0.429	9.089	0.000
	Brand culture	0.158	0.048	0.155	3.296	0.001
Control variable	Student	0.069	0.138	0.034	0.498	0.619
	Civil servant and public institution personnel	−0.272	0.170	−0.091	−1.601	0.110
	Enterprise personnel	0.009	0.147	0.004	0.058	0.954
	Businessman	−0.313	0.206	−0.081	−1.524	0.128
	Others	0				
	Convenience goods	−0.021	0.241	−0.007	−0.087	0.931
	Shopping goods	−0.206	0.288	−0.101	−0.713	0.476
	Specialty goods	−0.130	0.182	−0.057	−0.710	0.478
	Unsought goods	0				
	Gender	−0.113	0.093	−0.055	−1.212	0.226
	Monthly income	−0.101	0.093	−0.123	−1.089	0.277
*R*		0.619				
*R* ^2^		0.383				
Adjusted *R*^2^		0.359				
F		16.372				
Sig		0.000				

*The dependent variable is the service value*.

We obtained the linear regression equation for Model 7 as follows:

Service Value = 0.344 ^*^ Brand Character + 0.429 ^*^ Brand Value + 0.155 ^*^ Brand Culture +ε (Formula 7).

#### Regression Analysis of Experience Value to Consumers' Purchase Intention

Regression analysis was conducted with the experience value and each dimension as the independent variable in the regression equation and the consumer purchase intention as the dependent variable in the regression equation.

##### Regression Analysis of Experience Value to Consumers' Purchase Intention

From Table 9, we concluded that the standardized coefficient of experience value amounted to 0.560, which was a positive value with a significance level of 0.000. The results showed that the experience value can play a significant role in enhancing the consumers' purchase intention. Therefore, H3 has been verified.

**Table 9:** Regression analysis of experience value to consumers' purchase intention.

	**Model 8**	**Unstandardized coefficient**		**Standardized coefficient**	***t***	**Significance**
		**B**	**Standard error**	**Beta**		
Independent variable	(Constant)	1.006	0.378		2.659	0.008
	Experience value	0.686	0.058	0.560	11.912	0.000
Control variable	Student	0.046	0.111	0.027	0.412	0.681
	Civil servant and public institution personnel	0.063	0.135	0.025	0.465	0.642
	Enterprise personnel	0.248	0.119	0.125	2.087	0.038
	Businessman	0.491	0.165	0.154	2.977	0.003
	Others	0				
	Convenience goods	0.069	0.193	0.027	0.355	0.723
	Shopping goods	0.029	0.232	0.017	0.124	0.901
	Specialty goods	−0.029	0.147	−0.016	−0.198	0.843
	Unsought goods	0				
	Gender	0.049	0.075	0.029	0.660	0.510
	Monthly income	0.023	0.075	0.034	0.308	0.758
*R*		0.642				
*R* ^2^		0.413				
Adjusted *R*^2^		0.394				
F		22.399				
Sig		0.000				

*The dependent variable is the consumers' purchase intention*.

We obtained the linear regression equation for Model 8 as follows:

Consumers' Purchase Intention = 0.560 ^*^ Experience Value + 0.125 ^*^ Enterprise Personnel + 0.154 ^*^ Businessman + ε (Formula 8).

##### Regression Analysis of Each Dimension of Experience Value to Consumers' Purchase Intention

From Table 10, we concluded that the standardized coefficient of the four dimensions of experience value amounted to 0.229, 0.166, 0.245, and 0.228, respectively, which were all positive values with a significance level of 0.000. The results showed that the four dimensions of experience value can play a significant role in enhancing the service value. Therefore, H3a, H3b, H3c, and H3d have been verified.

**Table 10:** Regression analysis of each dimension of experience value to consumers' purchase intention.

	**Model 9**	**Unstandardized coefficient**		**Standardized coefficient**	***t***	**Significance**
		**B**	**Standard error**	**Beta**		
Independent variable	(Constant)	0.965	0.382		2.530	0.012
	Functional value	0.192	0.039	0.229	4.913	0.000
	Emotional value	0.128	0.036	0.166	3.546	0.000
	Social value	0.179	0.035	0.245	5.172	0.000
	Service value	0.189	0.038	0.228	4.961	0.000
Control variable	Student	0.051	0.111	0.030	0.459	0.646
	Civil servant and public institution personnel	0.090	0.138	0.036	0.651	0.515
	Enterprise personnel	0.255	0.120	0.129	2.131	0.034
	Businessman	0.510	0.167	0.160	3.049	0.002
	Others	0				
	Convenience goods	0.061	0.194	0.024	0.312	0.755
	Shopping goods	0.038	0.233	0.023	0.165	0.869
	Specialty goods	−0.025	0.147	−0.013	−0.168	0.866
	Unsought goods	0				
	Gender	0.051	0.075	0.030	0.686	0.493
	Monthly income	0.025	0.075	0.036	0.332	0.740
*R*		0.645				
*R* ^2^		0.415				
Adjusted *R*^2^		0.391				
F		17.273				
Sig		0.000				

*The dependent variable is the consumers' purchase intention*.

We obtained the linear regression equation for Model 9 as follows:

Consumers' Purchase Intention = 0.229 ^*^ Functional Value + 0.166 ^*^ Emotional Value + 0.245 ^*^ Social Value + 0.228 ^*^ Service Value + 0.129 ^*^ Enterprise Personnel + 0.160 ^*^ Businessman + ε (Formula 9).

#### Intermediary Role of Experience Value

The independent variable is set as each dimension of brand elements, the intermediary variable is set as each dimension of experience value, and the outcome variable is set as consumer purchase intention, with which different genders, occupations, income, and brand types as control variables to test the regression relationship of the above variables.

##### Functional Value

From Table 11, we noted that brand elements can impose a significant positive impact on consumers' purchase intention and functional value, whereas the functional value can also impose a significant positive impact on consumers' purchase intention. Subsequent to the addition of the intermediary variable, the results remained significant, whereas the regression coefficient of brand elements declined from 0.462 to 0.399 (*P* < 0.01), indicating the presence of the intermediary effect to some extent. Therefore, H4a has been verified.

**Table 11:** Intermediary role played by the functional value.

**Regression model**	**Variance analysis**				**Coefficient**	
	***R^**2**^***	**Adjusted *R^**2**^***	***F***	**Sig**.	***B***	**Sig**.
Brand elements → consumers' purchase intention	0.326	0.305	15.454	0.000	0.462	0.000
Brand elements → functional value	0.424	0.406	23.462	0.000	0.634	0.000
Functional value → consumers' purchase intention	0.249	0.226	10.580	0.000	0.329	0.000
Brand elements, functional value → consumers' purchase intention	0.332	0.309	14.374	0.000	0.399	0.000
					0.100	0.039

##### Emotional Value

Table 12 shows that brand elements can impose a significant positive impact on consumers' purchase intention and emotional value, whereas emotional value can also impose a significant positive impact on consumers' purchase intention. Subsequent to the addition of the intermediary variable, the results remained significant, whereas the regression coefficient of brand elements declined from 0.462 to 0.412 (*P* < 0.01), indicating the presence of the intermediary effect to some extent. Therefore, H4b has been verified.

**Table 12:** Intermediary role played by the emotional value.

**Regression model**	**Variance analysis**				**Coefficient**	
	***R^**2**^***	**Adjusted *R^**2**^***	***F***	**Sig**.	***B***	**Sig**.
Brand elements → consumers' purchase intention	0.326	0.305	15.454	0.000	0.462	0.000
Brand elements → emotional value	0.287	0.265	12.849	0.000	0.484	0.000
Emotional value → consumers' purchase intention	0.225	0.200	9.240	0.000	0.285	0.000
Brand elements, emotional value → consumers' purchase intention	0.334	0.311	14.506	0.000	0.412	0.000
					0.104	0.045

##### Social Value

From Table 13, brand elements can impose a significant positive impact on the purchase intention of consumers and the social value, whereas the social value can also impose a significant positive impact on the purchase intention of consumers. Subsequent to the addition of the intermediary variable, the results remained significant, whereas the regression coefficient of brand elements declined from 0.462 to 0.370 (*P* < 0.01), indicating the presence of the intermediary effect to some extent. Therefore, H4c has been verified.

**Table 13:** Intermediary role played by the social value.

**Regression model**	**Variance analysis**				**Coefficient**	
	***R^**2**^***	**Adjusted *R^**2**^***	***F***	**Sig**.	***B***	**Sig**.
Brand elements → consumers' purchase intention	0.326	0.305	15.454	0.000	0.462	0.000
Brand elements → social value	0.365	0.345	18.361	0.000	0.565	0.000
Social value → consumers' purchase intention	0.264	0.241	11.457	0.000	0.355	0.000
Brand elements, social value → consumers' purchase intention	0.343	0.321	15.124	0.000	0.370	0.000
					0.164	0.004

##### Service Value

From Table 14, brand elements can impose a significant positive impact on the purchase intention of consumers and the service value, whereas the service value can also impose a significant positive impact on the purchase intention of consumers. Subsequent to the addition of the intermediary variable, the results remained significant, whereas the regression coefficient of brand elements declined from 0.462 to 0.369 (*P* < 0.01), indicating the presence of the intermediary effect to some extent. Therefore, H4d has been verified.

**Table 14:** Intermediary role played by the service value.

**Regression model**	**Variance analysis**				**Coefficient**	
	***R^**2**^***	**Adjusted *R^**2**^***	***F***	**Sig**.	***B***	**Sig**.
Brand elements → consumers' purchase intention	0.326	0.305	15.454	0.000	0.462	0.000
Brand elements → service value	0.354	0.334	17.500	0.000	0.614	0.000
Service value → consumers' purchase intention	0.265	0.242	11.530	0.000	0.346	0.000
Brand elements, service value → consumers' purchase intention	0.341	0.318	14.969	0.000	0.369	0.000
					0.151	0.008

### Experimental Results of Research Hypotheses

Based on the aforementioned analysis, the experimental results of the research hypotheses are specified in Table 15.

**Table 15:** Experimental results of research hypotheses.

**Hypothesis No**.	**Hypothesis proposition**	**Verification of conclusion**
H1	Brand elements can impose a positive impact on the consumers' purchase intention	Established
H1a	Brand character can impose a positive impact on the consumers' purchase intention	Established
H1b	Brand value can impose a positive impact on the consumers' purchase intention	Established
H1c	Brand culture can impose a positively affects the consumers' purchase intention	Established
H2	Brand elements can impose a positive impact on the experience value	Established
H2a	Brand character, brand value and brand culture can, respectively, impose positive impacts on the functional value	Established
H2b	Brand character, brand value and brand culture can, respectively, impose positive impacts on the emotional value	Established
H2c	Brand character, brand value and brand culture can, respectively, impose positive impacts on the social value	Established
H2d	Brand character, brand value and brand culture can, respectively, impose positive impacts on the service value	Established
H3	Experience value can impose a positive impact on the consumers' purchase intention	Established
H3a	Functional value can impose a positive impact on the consumers' purchase intention	Established
H3b	Emotional value can impose a positive impact on the consumers' purchase intention	Established
H3c	Social value can impose a positive impact on the consumers' purchase intention	Established
H3d	Service value can impose a positive impact on the consumers' purchase intention	Established
H4a	Functional value can play an intermediary role in the relationship between brand elements and consumers' purchase intention	Established
H4b	Emotional value can play an intermediary role in the relationship between brand elements and consumers' purchase intention	Established
H4c	Social value can play an intermediary role in the relationship between brand elements and consumers' purchase intention	Established
H4d	Service value can play an intermediary role in the relationship between brand elements and consumers' purchase intention	Established

## Discussion

Through the above research, we found that the hypotheses proposed in this study have been verified, and the theoretical model of “brand elements - experience value - consumer purchase intention” has also been empirically supported. However, as mentioned earlier in this study, consumers' purchase intention is complex and will be affected by many factors. The brand element composition and experience value dimension proposed in this study are personal views obtained through a large number of studies. Compared with the research findings of global scholars in relevant fields during the same period, there are still many views worthy of reference. Lee et al. (2019) explored the impact of brand symbols on consumer behavior through the research on consumption patterns in the Internet environment and verified that brands have a significant positive impact on the perceived value by using Structural Equation Modeling (SEM). Kovanoviene et al. (2021) proposed the essence of marketing communication tools to help build a relationship between the consumer and the brand or company, so it can be considered to be a key tool for shaping the consumer loyalty. Diddi et al. (2019) expanded the meaning of brand elements: recyclability, durability, and repairability, and the positive impact of these characteristics on consumers' value orientation through the study of clothing consumption patterns. Vatamanescu et al. (2021) held the similar views. Compared with the period before and after the outbreak of COVID-19, it is proposed that consumers attach great importance to the social and environmental sustainability practices since the spread of the virus. Patak et al. (2021) put forward the concept of consumers' green purchase intention and pointed out that environmental concern, green lifestyle, and product knowledge are the main influencing factors. Similarly, Guo et al. (2021) put forward the concept of sustainable consumption intention. Thus, COVID-19 has profoundly changed the underlying factors of consumer buying behavior (Valaskova et al., 2021).

## Research Conclusion and Suggestions

### Conclusion

#### Brand Elements Can Impose a Positive Impact on the Consumers' Purchase Intention

Brand elements and three dimensions can impose a positive impact on the purchase intention of consumers. Moreover, in terms of the influencing degree, the dimensions of brand elements from strong to weak were brand value, brand character, and brand culture, respectively.

#### Brand Elements Can Impose a Positive Impact on the Experience Value

Brand elements can impose a significant role in enhancing the functional value. In terms of the influencing degree, the dimensions of brand elements from strong to weak were brand culture, brand character, and brand value, respectively.

Brand elements can play a significant role in enhancing emotional value. In terms of the influencing degree, the dimensions of brand elements from strong to weak include brand value, brand character, and brand culture, respectively. Emotional value serves as the bridge between consumers' mentality and products. Therefore, enterprises shall provide consumers with ample space for emotional sustenance, interest recognition, and imagination, so as to provide them with emotional comfort and enhance their loyalty to the brand.

Brand elements can play a significant role in enhancing social value. In terms of the influencing degree, the dimensions of brand elements from strong to weak include the brand character, brand value, and brand culture, respectively. Enterprises shall segment the target market and imbue the brand with features just like one's character. In addition, they shall highlight a specific personal style, level of consumption, and lifestyle of consumers, thereby enhancing the social value and other added values brought by the brand character.

Brand elements can play a significant role in enhancing the service value. In terms of the influencing degree, the dimensions of brand elements from strong to weak include brand value, brand character, and brand culture, respectively. Optimal brand image, higher popularity, and trustworthiness can bring greater service value to the brands.

#### Experience Value Can Impose a Positive Impact on the Consumers' Purchase Intention

Experience value can impose a positive impact on the purchase intention of consumers. In terms of the influencing degree, the dimensions of experience value from strong to weak include social value, functional value, service value, and emotional value, respectively. The results show that as consumers improve their income level and acquire a broader mental outlook, they have also upgraded their level of demand while paying more attention to the social value brought by the branded products. In addition, they attach greater importance to the service value and emotional value, providing a certain orientation for the in-depth development of the brand.

#### Intermediary Role Played by the Experience Value

Experience value can impose a positive impact on the brand elements and the purchase intention of consumers. The higher the experience value, the deeper the consumers' understanding of the brand, the higher their satisfaction with products and services, and the stronger their purchase intention.

### Suggestions

Therefore, enterprises should focus on brand value and brand character and supplement the efforts by promoting the brand culture in the meantime. This helps meet the respective needs of consumers at varying levels, and improve market competitiveness, so as to stimulate their purchase intention. At the same time, enterprises should carry out as several marketing activities as possible to improve consumers' brand perceived benefits, enhance consumers' brand perceived value, obtain consumers' brand perceived reputation, and make consumers form a positive attitude toward the brand. In addition, enterprises should use brand elements to accurately segment the target market and obtain the potential needs of consumer groups most suitable for the brand, thereby reducing the waste of ineffective brand communication.

## Study Limitations and Future Research Challenges

### Study Limitations

In the composition of the number of samples selected in this study, the age of the subject sample is relatively young, and the grasp-of-the-fit between the brand and itself may not be accurate enough, which may have some impact on the research conclusion. In addition, this paper focuses on the impact of brand elements on purchase intention from a macro perspective. The differences between industry and brand are limited by the survey samples and are not detailed enough. It is possible that the relationship between various variables is not thoroughly explored.

### Practical Implications

Enterprises are promoted to deepen their awareness of the importance of improving consumers' experience value in the process of brand construction, so as to promote and guide consumers' cultural identity, character identity, and value identity of the brand, thereby enhancing their purchase intention.

### Future Research Challenges

Combined with the influence of COVID-19 on consumer cognition, we can further complement and improve the connotation of brand elements. At the same time, in order to improve the accuracy of survey target selection, the scope of the sample distribution should be continuously expanded in follow-up research. In addition, the industry and brand can be further refined in the future. For a certain industry or brand, various factors can be fully considered to explore the relationship between brand elements, experience value, and consumers' purchase intention, so as to increase the accuracy of the data analysis.

## Data Availability Statement

The original contributions presented in the study are included in the article/Supplementary Material, further inquiries can be directed to the corresponding author/s.

## Ethics Statement

The studies involving human participants were reviewed and approved by Tianjin University of Commerce. The patients/participants provided their written informed consent to participate in this study.

## Author Contributions

YZ contributed to the conception and design of the study and wrote the first draft of the manuscript. MH organized the database, performed the statistical analysis, and wrote sections of the manuscript. All authors contributed to manuscript revision and read and approved the submitted version.

## Funding

This study was supported by the Research on Customer Stratification Management Based on Cost to Service Measurement in the Internet Era [Philosophy and Social Science Project (No. TJGL18-036), Tianjin, China] and Research on the Impact of Venture Capital and Organizational Culture on Start-up Enterprises [Major Social Science Projects of Tianjin Education Commission (No. 2017JWZD30), Tianjin, China], and Research on customer stratification method and its application based on machine learning (2021 Tianjin Graduate Scientific Research Innovation Project (2021YJSS272), Tianjin, China).

## Conflict of Interest

The authors declare that the research was conducted in the absence of any commercial or financial relationships that could be construed as a potential conflict of interest.

## Publisher's Note

All claims expressed in this article are solely those of the authors and do not necessarily represent those of their affiliated organizations, or those of the publisher, the editors and the reviewers. Any product that may be evaluated in this article, or claim that may be made by its manufacturer, is not guaranteed or endorsed by the publisher.
