# Supplementary material for: The Impact Imposed by Brand Elements of Enterprises on the Purchase Intention of Consumers—With Experience Value Taken as the Intermediary Variable
Source: Front Psychol. 2022 Jun 9;13:873041. doi: 10.3389/fpsyg.2022.873041 (PMC9220800; doi:10.3389/fpsyg.2022.873041)
Supplement: Supplementary file 1 [file Table_1.docx]

Supplement Table 1 Measurement Scale of Brand Elements

| Variable | No. | Measurement question | Source |
| --- | --- | --- | --- |
| Brand character | A11 | I like the brand with a unique character | Dong (2019) |
|  | A12 | The brand character is quite similar to mine |  |
|  | A13 | The brand character is exactly what I would like to have |  |
| Brand value | A21 | The brand image can impose an impact on my purchasing preference | Keller (2003)  Altaf, Iqbal, Mohd Mokhtar and Sial (2017) |
|  | A22 | I purchase the products of a specific brand given that it enjoys great reputation |  |
|  | A23 | I believe the brand is trustworthy |  |
|  | A24 | I have acquired a lot of brand knowledge |  |
| Brand culture | A31 | I am moved by the beautiful vision of the brand | Huang (2016) |
|  | A32 | I like a certain brand on the grounds that I identify with its values |  |
|  | A33 | The spiritual symbols that the brand conveys resonate with me |  |
|  | A34 | I value the developmental history of the brand |  |
|  | A35 | The stories of the brand impress me |  |
