# Supplementary material for: The Impact Imposed by Brand Elements of Enterprises on the Purchase Intention of Consumers—With Experience Value Taken as the Intermediary Variable
Source: Front Psychol. 2022 Jun 9;13:873041. doi: 10.3389/fpsyg.2022.873041 (PMC9220800; doi:10.3389/fpsyg.2022.873041)
Supplement: Supplementary file 2 [file Table_2.docx]

Supplement Table 2 Measurement Scale of Experience Value

| Variable | No. | Measurement question | Source |
| --- | --- | --- | --- |
| Functional value | B11 | The branded products feature sophisticated workmanship | Sweeney and Soutar (2001)  Cheng (2010) |
|  | B12 | The branded products feature powerful function |  |
|  | B13 | The branded products feature optimal effect of use |  |
|  | B14 | The branded products feature better quality than similar products |  |
| Emotional value | B21 | This brand makes me feel comfortable | Holbrook (2006) |
|  | B22 | The branded products can bring me happiness |  |
|  | B23 | The branded products are the ones I like |  |
|  | B24 | Using the product has become a pleasure for me |  |
| Social value | B31 | The branded products can improve my social level | Zou, Wang, Zhao, and Wang (2007) |
|  | B32 | The branded products can enhance others’ perception of me |  |
|  | B33 | The branded products can help me gain more social recognition |  |
|  | B34 | The branded products allow me to become more integrated into certain occasions |  |
| Service value | B41 | Optimal shopping environment makes me satisfied | Ulaga and Chacour (2001)  Ai (2017) |
|  | B42 | The sales staff have an optimal attitude and high quality of service |  |
|  | B43 | The after-sales service of the brand is complete with all that is desired |  |
|  | B44 | The online sales of good brands make me feel quite convenient |  |
