# Supplementary material for: The Impact Imposed by Brand Elements of Enterprises on the Purchase Intention of Consumers—With Experience Value Taken as the Intermediary Variable
Source: Front Psychol. 2022 Jun 9;13:873041. doi: 10.3389/fpsyg.2022.873041 (PMC9220800; doi:10.3389/fpsyg.2022.873041)
Supplement: Supplementary file 3 [file Table_3.docx]

Supplement Table 3 Measurement Scale of the Purchase Intention of Consumers

| Variable | No. | Measurement question | Source |
| --- | --- | --- | --- |
| Consumer purchase intention | C1 | I wish to buy certain products of the brand | Anckar and D’Incau (2002) |
|  | C2 | I would recommend the products of the brand to my friends and relatives |  |
|  | C3 | I prefer to buy the products of the brand again instead of other similar products |  |
