# Supplementary material for: The Impact Imposed by Brand Elements of Enterprises on the Purchase Intention of Consumers—With Experience Value Taken as the Intermediary Variable
Source: Front Psychol. 2022 Jun 9;13:873041. doi: 10.3389/fpsyg.2022.873041 (PMC9220800; doi:10.3389/fpsyg.2022.873041)
Supplement: Supplementary file 4 [file Table_4.docx]

Supplement Table 4 Reliability Analysis of the Scales

| Variable | Dimension | Number of questions | *Cronbach’s Alpha* | *Cronbach’s Alpha* |
| --- | --- | --- | --- | --- |
| Questionnaire | | 31 | 0.925 | |
| Brand elements | Brand character | 3 | 0.869 | 0.832 |
|  | Brand value | 4 | 0.915 |  |
|  | Brand culture | 5 | 0.914 |  |
| Experience value | Functional value | 4 | 0.879 | 0.854 |
|  | Emotional value | 4 | 0.890 |  |
|  | Social value | 4 | 0.897 |  |
|  | Service value | 4 | 0.898 |  |
| Consumers’ purchase intention | | 3 | 0.816 | 0.816 |
