# Supplementary material for: The Impact Imposed by Brand Elements of Enterprises on the Purchase Intention of Consumers—With Experience Value Taken as the Intermediary Variable
Source: Front Psychol. 2022 Jun 9;13:873041. doi: 10.3389/fpsyg.2022.873041 (PMC9220800; doi:10.3389/fpsyg.2022.873041)
Supplement: Supplementary file 5 [file Table_5.docx]

Supplement Table 5 KMO and Bartlett’s Test of Brand Elements

| $KMO$ measurement of sampling adequacy | | 0.845 |
| --- | --- | --- |
| Bartlett’s test | Approx. Chi-square | 2588.772 |
|  | Degree of freedom | 66 |
|  | Significance | 0.000 |
