# Supplementary material for: The Impact Imposed by Brand Elements of Enterprises on the Purchase Intention of Consumers—With Experience Value Taken as the Intermediary Variable
Source: Front Psychol. 2022 Jun 9;13:873041. doi: 10.3389/fpsyg.2022.873041 (PMC9220800; doi:10.3389/fpsyg.2022.873041)
Supplement: Supplementary file 6 [file Table_6.docx]

Supplement Table 6 Rotating Component Matrix of Brand Element Scale

| Variable | Question | Factor load | | |
| --- | --- | --- | --- | --- |
|  |  | Factor 1 | Factor 2 | Factor 3 |
| Brand character |  | 0.891 |  |  |
|  |  | 0.903 |  |  |
|  |  | 0.861 |  |  |
| Brand value |  | 0.090 | 0.879 |  |
|  |  |  | 0.901 |  |
|  |  |  | 0.887 |  |
|  |  |  | 0.878 |  |
| Brand culture |  |  |  | 0.866 |
|  |  |  |  | 0.887 |
|  |  |  |  | 0.851 |
|  |  |  |  | 0.887 |
|  |  |  |  | 0.791 |
