# Supplementary material for: The Impact Imposed by Brand Elements of Enterprises on the Purchase Intention of Consumers—With Experience Value Taken as the Intermediary Variable
Source: Front Psychol. 2022 Jun 9;13:873041. doi: 10.3389/fpsyg.2022.873041 (PMC9220800; doi:10.3389/fpsyg.2022.873041)
Supplement: Supplementary file 8 [file Table_8.docx]

Supplement Table 8 Rotating Component Matrix of Experience Value Scale

| Variable | Question | Factor load | | | |
| --- | --- | --- | --- | --- | --- |
|  |  | Factor 1 | Factor 2 | Factor 3 | Factor 4 |
| Functional value | B11 |  |  |  | 0.868 |
|  | B12 |  |  |  | 0.876 |
|  | B13 |  |  |  | 0.870 |
|  | B14 |  |  |  | 0.762 |
| Emotional value | B21 |  |  | 0.878 |  |
|  | B22 |  |  | 0.876 |  |
|  | B23 |  |  | 0.884 |  |
|  | B24 |  |  | 0.773 |  |
| Social value | B31 |  | 0.866 |  |  |
|  | B32 |  | 0.861 |  |  |
|  | B33 |  | 0.871 |  |  |
|  | B34 |  | 0.835 |  |  |
| Service value | B41 | 0.788 |  |  |  |
|  | B42 | 0.905 |  |  |  |
|  | B43 | 0.882 |  |  |  |
|  | B44 | 0.881 |  |  |  |
