# Supplementary material for: The Impact Imposed by Brand Elements of Enterprises on the Purchase Intention of Consumers—With Experience Value Taken as the Intermediary Variable
Source: Front Psychol. 2022 Jun 9;13:873041. doi: 10.3389/fpsyg.2022.873041 (PMC9220800; doi:10.3389/fpsyg.2022.873041)
Supplement: Supplementary file 9 [file Table_9.docx]

Supplement Table 9 KMO and Bartlett Test of Consumers’ Purchase Intention

| *KMO* measurement of sampling adequacy | | 0.704 |
| --- | --- | --- |
| Bartlett’s test | Approx. Chi-square | 350.060 |
|  | Degree of freedom | 3 |
|  | Significance | 0.000 |
