# Supplementary material for: The Impact Imposed by Brand Elements of Enterprises on the Purchase Intention of Consumers—With Experience Value Taken as the Intermediary Variable
Source: Front Psychol. 2022 Jun 9;13:873041. doi: 10.3389/fpsyg.2022.873041 (PMC9220800; doi:10.3389/fpsyg.2022.873041)
Supplement: Supplementary file 10 [file Table_10.docx]

Supplement Table 10 Exploratory Factor Analysis of Consumers’ Purchase Intention Scale

| Variable | Question | Factor load 3 |
| --- | --- | --- |
| Consumers’ purchase intention | C1 | 0.869 |
|  | C2 | 0.819 |
|  | C3 | 0.878 |
